# Exercise therapy versus surgery for lumbar spinal stenosis: A systematic review and meta-analysis

**DOI:** 10.12669/pjms.344.14349

**Published:** 2018

**Authors:** Zhuomao Mo, Renwen Zhang, Minmin Chang, Shujie Tang

**Affiliations:** 1 Zhuomao Mo, School of Chinese Medicine, Jinan University, Guangzhou, Guangdong Province, 510632, China; 2 Renwen Zhang, School of Chinese Medicine, Jinan University, Guangzhou, Guangdong Province, 510632, China; 3 Minmin Chang, School of Chinese Medicine, Jinan University, Guangzhou, Guangdong Province, 510632, China; 4 Shujie Tang, School of Chinese Medicine, Jinan University, Guangzhou, Guangdong Province, 510632, China

**Keywords:** 36 Items Short Form Health Survey, Exercise, Lumbar spinal stenosis, Meta-analysis, Oswestry Disability Index, Surgery

## Abstract

**Objective:**

To compare the effectiveness of exercise therapy with surgery for lumbar spinal stenosis.

**Methods:**

Five English databases PubMed, the Cochrane Library, Web of science, OVID and PEDro database were searched for randomized controlled trials comparing surgical procedures with exercise therapy for lumbar spinal stenosis. Information on patients, study design, inclusion criteria, intervention and follow-up, outcomes, treatment details and adverse events were extracted. Meta-analysis was performed using Review Manager Version 5.3.

**Results:**

Two randomized controlled trials and one mixed design trial with a total of 897 patients were included. The pooled results showed a significant difference between exercise and surgery in Oswestry Disability Index at two years (MD= 3.85, 95%CI: 0.48 to 7.22; P=0.03), but no significant difference at six months (MD= 2.18, 95%CI: -2.80 to 7.17; P=0.39) and one year (MD= 4.26, 95%CI: -1.79 to 10.32; P=0.17). In terms of physical function of 36 Items Short Form Health Survey, there were no significant differences between exercise and surgery at six months (MD= -2.23, 95% CI: -7.46 to 2.99; P=0.40), one year (MD= -2.17, 95% CI: -7.44 to 3.10; P=0.42) and two years (MD= -0.67, 95% CI: -6.16 to 4.82; P=0.81).

**Conclusion:**

In brief, the current evidence demonstrated a trend that exercise therapy had a similar effect for lumbar spinal stenosis compared with decompressive laminectomies. However, for the small sample size and low methodology quality of the included trials, some rigorously designed and large-scaled RCTs need to be performed to confirm the conclusion.

## INTRODUCTION

Lumbar spinal stenosis (LSS) is one of the most prevalent and disabling conditions in people older than 65 years, which may lead to pain in low back, buttocks or lower extremities as well as neurogenic claudication^1^, affecting the quality of life in patients adversely. Considering the coming of aging society, more people may suffer from LSS and it is critical to pay a high attention to its therapy. LSS can be treated using surgical or nonsurgical modalities, given that the slow process, degenerative condition and operative complications of LSS, conservative treatment should be a primary option.^2^

As one of the conservative modalities, exercise therapy can play an important role in the treatment of LSS. In a study of forty-five patients with LSS, Ahmet found that the leg pain and disability scores significantly decreased in patients treated using three-week therapeutic exercises compared with those without treatment.^3^ In another study of fifty patients, Homayouni^4^ found that aquatic exercises provided greater short-term improvement in visual analog scale (VAS) and six-minute walk test than conventional physical therapy. Some other studies also had the similar conclusions, demonstrating the satisfying efficacy of exercise therapy.^5^,^6^

In addition, patients with severe spinal stenosis are usually treated using surgical procedures, and subsequently it is important to evaluate the effectiveness of exercise therapy versus surgery. In a systematic review, Jarrett^7^ suggested that lumbar decompressive surgery was more effective than land based exercise for LSS. However, in his study only one trial compared directly the effectiveness of exercise with surgery, and the comparison between other studies were performed using percentage change. In recent years, more studies have been published to compare the effectiveness of exercise therapy with surgery for LSS, some of which demonstrated a different viewpoint. In a systematic review, Macedo^8^ concluded that surgery had no advantages in Oswestry Disability Index (ODI) six months and one year after treatment when compared with physical therapy including exercises. In a study of 169 participants, Delitto suggested that surgery presented with similar effects for patients with LSS as exercise therapy.^9^ Subsequently, we believe that an updated systematic review and meta-analysis is needed to reevaluate the effectiveness of exercise therapy versus surgery for LSS.

Therefore, we performed this systematic review and meta-analysis, and our purpose was to evaluate the evidence of the effectiveness of exercise therapy versus surgery.

## METHODS

### Data sources

A medical literature search was carried out in the following databases from their inception through June 15, 2017: PubMed, the Cochrane Library, Web of science, OVID and PEDro database. The language of these studies was restricted in English. The searching was performed using medical subject headings (MeSH) and keywords. The search terms were (Lumbar stenosis or intermittent claudication or canal stenosis) and (exercises or exercise therapy or general exercise or traditional exercise or conventional exercise or specific exercise or pilates or resistance training or rehabilitation or bicycling or lumbar stability or core stability or transversus or abdominis or multifidus) and (randomized controlled trial or random allocation or clinical trials or double-blind method or single-blind method) and (lumbar or lumbar spine or lumbar vertebra). Two investigators performed the search independently to confirm the consistency of the search. Two investigators independently reviewed the titles and abstracts of articles and chose the potential ones, then checked full texts of the selected articles according to inclusion and exclusion criteria, in which the third person checked the divergent articles.

### Inclusion criteria

Trials were included based on the following criteria:

1. Randomized controlled trials (RCTs)
2. Trials comparing surgical procedure with exercise therapy of any types for LSS
3. Trials in which exercise therapy was the sole treatment or part of treatment package in exercise group, while surgery was the sole treatment in surgery group
4. Trials published in English
5. Trials in which LSS was diagnosed by symptoms, signs and imaging examination

The exclusion criteria consisted:

1. Non-RCTs
2. Literature review
3. Duplicate studies
4. Case reports or expert opinions
5. Studies comparing different types of exercise therapies or surgical procedures
6. Studies without integrated data

### Data extraction

Two reviewers independently participated in the data extraction, and collected the following information:

1. Basic characteristics, including author name, study type and design, sample size, inclusion criteria, intervention measures, outcomes, treatment duration and adverse events.
2. Outcome measurements
3. Follow-up period.

### Quality assessment

Quality assessment was independently performed according to the Cochrane Risk of Bias Tool by two reviewers. Disagreements were checked by the third reviewer. Risk of bias included the items of random sequence generation, allocation concealment, blinding of participants and personnel, blinding of outcome assessment, incomplete outcome data, selective reporting and other bias, in which blinding of participants and personnel were disregarded, as it was not applicable when comparing surgery with exercise therapies.

### Statistical analysis

A meta-analysis was carried out using Review Manager Version 5.3. Continuous variables were analyzed using the mean differences (MD) with its 95% confidence interval (CI), and the dichotomous data using the risk ratio (RR) and its 95% CI. The heterogeneity was quantified by calculating the value of I², and I^2^ ≥ 50% indicated a substantial level of heterogeneity. Fixed effects model was used when there was no significant heterogeneity between trials, otherwise random effects model was employed. P<0.05 was regarded as significant level.

## RESULTS

### Identification of relevant studies

**Fig.1:**
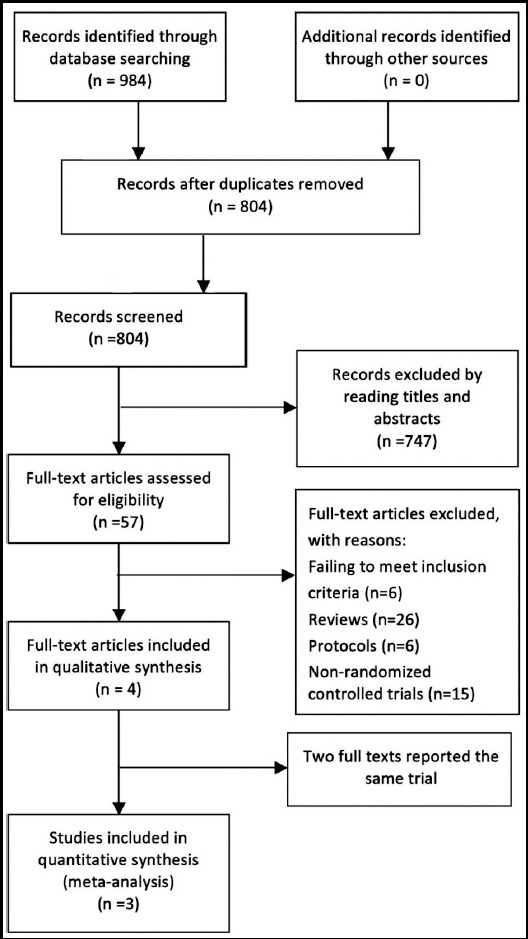
The flow chart of selecting studies.

984 articles were identified in our initial search, from which 180 were excluded for duplications, 747 were excluded by reading titles and abstracts. The full text of the remaining 57 articles were obtained to check eligibility, in which 6 were excluded because of failing to meet the inclusion criteria, 26 were excluded for review articles, six were excluded for protocols and 15 were excluded for non-RCTs. In the remaining 4 full texts, two reported the same trial with different follow-up period, so only three trials^9^-^11^ were included in our final analysis. Fig.1 shows the selection process for relevant studies.

### Characteristics of the included trials

The included trials were all published in English. The trials involved 897 patients who included 504 males and 393 females, and the sample sizes ranged from 94 to 654 cases. The baseline characteristics of included studies are presented in Table-I. Of the three trials, two were RCTs^9^,^10^ and one was mixed-design study^11^ including a RCT cohort and an observational cohort. In this study, only the data in RCTs were pooled for meta-analysis. All trials employed exercise therapy as primary interventions. In addition to exercise therapy, nonsteroidal anti-inflammatory drugs and education were used in two trials.^10^,^12^ All the surgeries were decompressive laminectomies. ODI was used as outcome measurement in all the 3 trials, physical function of SF-36 was used in two trials.^9^,^12^ All the trials reported side effects.

**Table-I:** Baseline characteristics of the included trials.

Study Type	Participants	Intervention measures	Outcome measurement	No. of treatment session	Program duration	Follow-up	Side effect	Study design
Weinstein^11^,^12^ et al.	654 patients	Exercises therapy + other treatment vs. decompressive laminectomy.	SF-36, ODI.	Not stated	Not stated	Follow-up:6 weeks, 3 months, 6 months, 1 year, 2 years, 3 years, 4 years	Side effect occurred in surgery group, including transfusions and dural tear.	Mixed design (Randomized trial and observational Cohort study).
Malmivaara^10^et al.	94 patients.	Exercises + other treatment vs. decompressive laminectomy.	ODI, walking capacity	1-3 times/week	2 years	Follow-up: 6, 12 and 24 months.	Side effect occurred in surgery group, including dural sac lesion, misplaced transpedicular screw, neural dysfunction, misjudgment of stenotic level, and respiratory distress.	Randomized controlled trial
Delitto^9^ et al.	169 patients.	Exercises vs. decompressive laminectomy.	SF-36, ODI.	2 times/week	6 weeks	Follow-up:10 weeks, 6 months, 12 months, 24 months	Surgery-related complications including delay in wound healing, surgical site infection, respiratory tract infection, blood loss, and other complications; Exercise and physical therapy-related complications is worsening symptoms.	Randomized controlled trial

Note: ODI: Oswestry Disability Index, SF-36: 36-Item Short-Form Health Survey.

### Quality assessment

The risk of bias assessment was performed based on the Cochrane criterion. The patients in two RCTs^9^,^10^ were randomly assigned to exercise or surgery group using network stochastic system. In the mixed-design study, the patients in RCT cohort were randomly assigned, but it didn’t mention the randomization method in details. In allocation concealment, one trial^13^ used the opaque and sealed envelopes, one trial^10^ received treatment allocation by phone and one trial^12^ didn’t state. In addition, blinding of the participants was not carried out because of the informed consent process, and blinding of judger wasn’t performed because it didn’t affect the final results of the trials. All trials^10^,^12^,^13^ reported entire outcome data.

### Meta-analyses results

**Fig.2:**
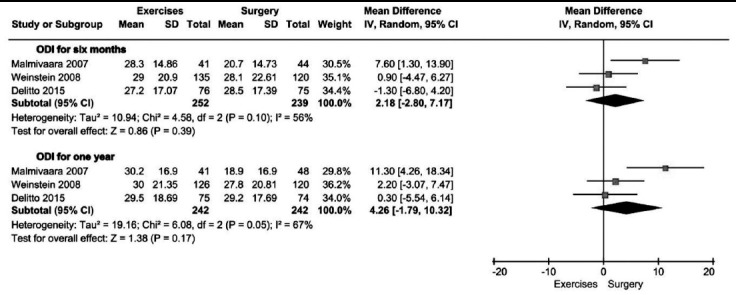
Exercise versus surgery in ODI at six months and one year.

**Fig.3:**
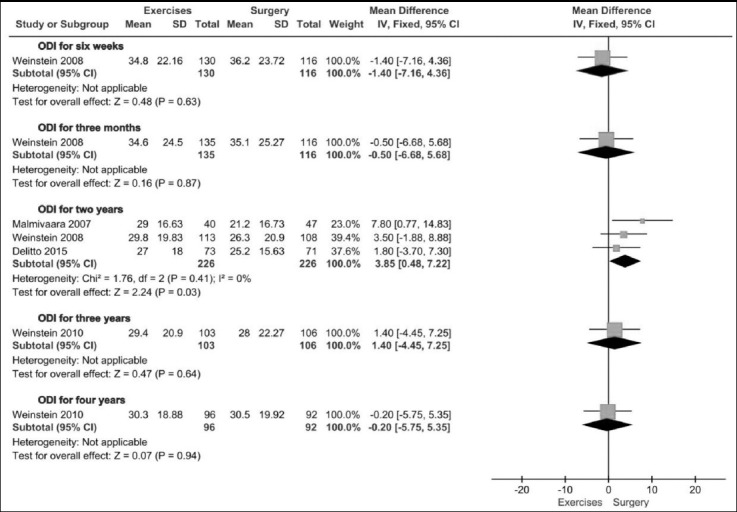
Exercise versus surgery in ODI at six weeks, three months, two years, three years and four years.

All the trials^10^,^12^,^13^ used ODI as primary outcome measurement. As illustrated in Fig.2 and Fig.3, the heterogeneity for ODI at six months and one year was high, but at other follow-up time point was low. The pooled results showed no significant difference between exercise and surgery in ODI at six months (491 patients. MD= 2.18, 95%CI: -2.80 to 7.17; P=0.39) and one year (484 patients. MD= 4.26, 95%CI: -1.79 to 10.32; P=0.17) (Fig.2), but a significant difference at two years (452 patients. MD= 3.85, 95%CI: 0.48 to 7.22; P=0.03) (Fig.3), In addition, Weinstein’s study indicated no significant difference in ODI between exercise and surgery group at six weeks, three months, three years and four years (Fig.3).

**Fig.4:**
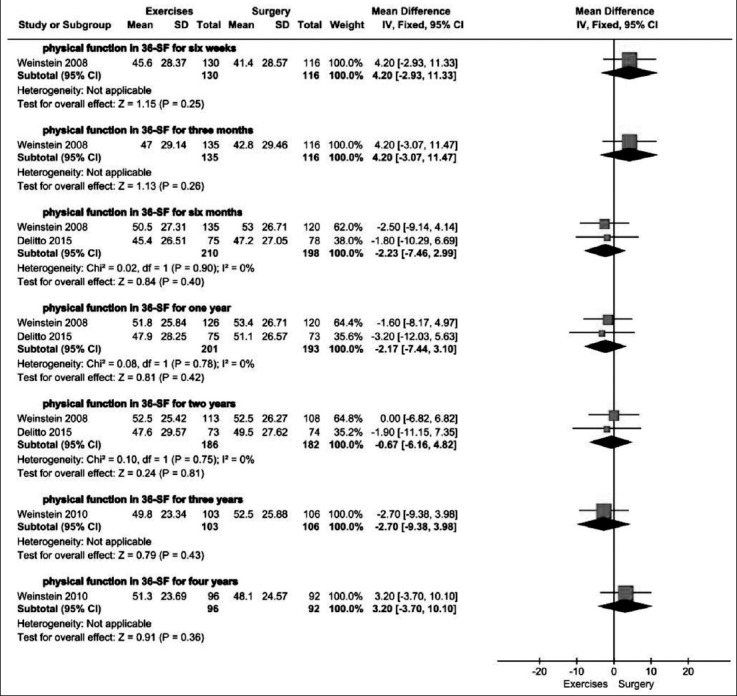
Exercise versus surgery in physical function of 36-SF at six weeks, three months, six months, one year, two years, three years and four years.

Two trials^12^,^13^ used physical function of 36-SF as outcome measurement. As illustrated in Fig.4, the heterogeneity was low, so a fixed-effects model was employed. The pooled data showed no significant difference in physical function of 36-SF between exercise therapy and surgery group at six months (408 patients. MD=-2.23, 95% CI:-7.46 to 2.99; P=0.40), one year (394 patients. MD= -2.17, 95% CI: -7.44 to 3.10; P=0.42) and two years (368 patients. MD=-0.67, 95% CI: -6.16 to 4.82; P=0.81). At the same time, Weinstein’s study also demonstrated no significant difference between exercise and surgery group at six weeks, three months, three years and four years (Fig.4).

All trials reported side effects. In Weinstein’s trial^11^, 9% patients suffered from dural tear, 10% required transfusion during surgery and 5% required postoperatively. Malmivaara’s trial^10^ also reported side effects in surgery group, in which perioperative complications included dural sac lesion in seven cases and misplaced transpedicular screw in one case, and postoperative complications included neural dysfunction in 1 case, misjudgment of stenotic level in one case and respiratory distress in one case. In Delitto’s trial^9^, 33 cases suffered from surgery-related complications, including delay in wound healing, surgical site infection, respiratory tract infection, blood loss and other complications, and nine cases suffered from worsening symptoms in exercise group.

## DISCUSSION

In this systematic review and meta-analysis, the efficacy of exercise therapy versus surgery for LSS was evaluated, which may help physicians better make treatment strategies in the treatment of LSS.

In the included trials, ODI and physical function of SF-36 were primary outcome measurements. The pooled results in physical function of SF-36 demonstrated no significant difference between exercise therapy and surgery group at every follow-up time point. Also, at most of follow-up time points the pooled results of ODI showed no significant differences. However, at two years the pooled result of ODI showed a favorable effectiveness of surgery. The difference may be attributed to Malmivaara’s trial, in which most of outcome measurements presented with a better efficacy of surgery.^10^

In terms of the mechanism of exercise therapy for LSS, it may be attributed to its effect on lumbar alignment. Exercises can increase the activation of paravertebral muscles, improve the stability and coordination of lumbar spine, improve lumbar lordosis angle and adjust the lumbar alignment and subsequently it can result in the relief of nerve compression, and the symptoms including pain and disability are improved in patients with LSS.^2^,^14^,^15^ Compared with exercise therapy, lumbar decompressive laminectomies can relieve pain immediately, but it can’t strengthen the power of muscles and flexibility of joints. In addition, surgical procedures may damage the paravertebral muscles, decrease the muscle power and adversely affect the lumbar alignment. Consequently, in the current study, most of outcome measurements demonstrated a favorable effect of exercise therapy. Although we can’t obtain a completely consistent conclusion, we can find a trend, indicating that exercise therapy has similar efficacy as surgery.

### Limitations of the study

First, the included trials evaluating exercise therapy interventions versus surgery for patients with LSS were few, and one trial lacked details in the domains of randomization sequence generation and allocation concealment, and one trial had small sample size, which may affect the quality of evidence and strength of recommendation. Second, exercise therapy programs, including session number, session duration, program duration and other details, were not provided in some trials, which may affect the interpretation of study results and replication in future studies. Third, in the analysis of ODI at six months and one year, the heterogeneity is high, which may be attributed to the difference of intervention measures, such as different types of exercise therapies or physical therapies were used in trials.

In view of the above-mentioned limitations, more RCTs evaluating the effect of exercise therapy versus surgery for LSS with large-scaled sample size should be performed to provide higher quality evidence. Moreover, the methodology quality of trials should be paid high attention to, and the details of interventions such as exercise type, time spent on intervention and program duration should be provided in future trials. In addition, considering the heterogeneity between exercise therapy and some cointerventions, in future RCTs exercise therapy should be used as unique intervention measure in exercise group.

## CONCLUSION

In brief, the current evidence demonstrated a trend that exercise therapy had a similar effect for LSS as decompressive laminectomies. However, as the quantity was small and methodology quality was low in the included trials, some rigorously designed and large-scaled RCTs need to be performed to confirm the current conclusion.

### Author`s Contribution

**SJT** conceived, designed and did statistical analysis & editing of manuscript.

**ZMM, RWZ, MMC** did data collection and manuscript writing.

**SJT** did review and final approval of manuscript.
